# Provider Recommendations Are Associated with Cancer Screening of Transgender and Gender-Nonconforming People: A Cross-Sectional Urban Survey

**DOI:** 10.1089/trgh.2019.0083

**Published:** 2020-06-08

**Authors:** Mandi L. Pratt-Chapman, Adam R. Ward

**Affiliations:** ^1^ Department of Clinical Research and Leadership, GW Cancer Center, School of Medicine and Health Sciences, The George Washington University, Washington, District of Columbia, USA.; ^2^ Department of Microbiology, Immunology, and Tropical Medicine, School of Medicine and Health Sciences, The George Washington University, Washington, District of Columbia, USA.

**Keywords:** adherence, cancer screening, gender nonconforming, provider recommendations, transgender

## Abstract

**Purpose:** This study examined the relationship between clinician recommendation and receipt of cancer screenings among a transgender and gender-nonconforming (TGNC) sample (*n*=58).

**Methods:** Respondents self-identified as TGNC, age 40+ years, and residents of the Washington, D.C. area. Odds ratios were calculated to compare provider-recommended with received screenings. An open-text question asked for recommendations to improve screening experiences.

**Results:** Provider recommendations were associated with screenings for breast, colorectal, prostate, lung, and anal cancer. Respondents cited interpersonal skills, affirming language, and clear information as important health care provider characteristics.

**Discussion:** Participants reported being more likely to be screened if a provider recommended one regardless of evidence from current published guidelines.

**Conclusion:** Gender identity, anatomy, and hormone exposure are critical elements that should be collected in future cancer screening research to build a stronger evidence base for provider recommendations based on population-level and individual-level risks of TGNC people.

## Introduction

Transgender and gender-nonconforming (TGNC) people have unique health care needs due to gender-affirming hormonal therapy and/or surgical interventions. Health care curricula in professional schools are lacking, and most health care professionals receive little training to provide clinically and culturally appropriate health care to TGNC patients.^1–4^ Yet, the TGNC community is at a higher risk for some cancers. From a population perspective, sexual and gender minorities have higher rates of alcohol and tobacco use.^5^ HPV-related cancers are associated with exposure: thus individuals, regardless of transgender status, who have oral and/or anal-receptive sex are at risk for oropharynx and anal cancers, respectively.^5^ Breast cancer risks for transgender women with longitudinal exposure to estrogen are reported to be greater than cisgender women but less than cisgender men.^6^ Cervical cancer risks for transgender men are sometimes erroneously assumed by providers to be lower than for cisgender women.^7^

Cancers are most treatable when cancer screening is guideline driven.^8^ However, currently only consensus-based guidelines exist to inform cancer screening recommendations for TGNC people.^9^ The risks for breast, cervical, and prostate cancer for TGNC people are not known, given the varying levels and types of hormone exposure among TGNC people and a critical lack of research to inform clinical practice for this population. Consensus-based guidelines recommend that transmasculine people with sufficient breast tissue and transfeminine people exposed to 5 or more years of estrogen and older than 50 years receive mammography consistent with guidelines for cisgender women.^9^ Anyone older than 21 years with a cervix is at risk for cervical cancer and should be screened based on guidelines for cisgender women.^5,9^ All people older than 50 years should be screened for colorectal cancer,^5,10^ and anyone older than 55 years with a 30 pack-year history of smoking should be referred for lung cancer screening.^5,11^ Anal cancer screening recommendations are based on risks associated with sexual practices. Based on the European Society for Medical Oncology guidelines, people who have a history of anal-receptive sex should be screened.^12^ The U.S. Preventive Services Task Force (USPSTF) does not currently have a clinical practice guideline for anal cancer screening. Furthermore, the USPSTF considers evidence for oral and skin screening inconclusive.^13,14^

Despite a call to action from the National Institutes of Health^15^ and the American Society of Clinical Oncology,^16^ cancer research to inform the care of TGNC patients has lagged. In fact, no known studies examining TGNC cancer screening experiences exist to date. The primary aim of this study was to examine the relationship between clinician recommendations for and participant receipt of cancer screenings in a TGNC sample in the Washington, D.C. metropolitan area based on participant self-report. The research question was as follows: Are TGNC more likely to receive a cancer screening if a provider recommends the screening versus no provider recommendation?

## Methods

### Participants and procedures

The George Washington University Institutional Review Board (IRB) determined this study to be exempt from IRB review under Department of Health and Human Services category 2 (IRB No. NCR1911213). Participants were provided information on the study before completing a survey. The following statement was included: “Your willingness to participate is implied if you complete a survey.” Participants were asked questions about age, self-identification along the transgender spectrum, and residency to ensure eligibility before completing the survey. Respondents were eligible to take the online survey at an in-person event if they reported being TGNC, age 40 years or older, and a resident of the Washington, D.C. metropolitan area. A fourth screening question asked participants to provide their true experiences when completing the survey. Data were collected on electronic tablets at three local transgender-affirming community events from April to July 2019. Responses were saved within the secure, cloud-based REDCap database and exported to SPSS 24 (Armonk, NY) and SAS v9.4 (Cary, NC) for analysis.

### Survey measures

Following the four screening questions, the survey included 50 items that asked participants about which cancer screenings had been recommended to them by providers, which cancer screenings the respondent had received, and how long it had been since receiving each screening (see Supplemental Appendix S1). Additional questions asked about age, race/ethnicity, sex assigned at birth, exposure to hormonal therapy, transgender-affirming surgical procedures, regular health care provider, and personal and family history of cancer. Respondents were asked about receipt of anal, breast, cervical, colorectal, lung, oral, and skin cancer screenings based on skip logic using sex assigned at birth and gender-affirming surgeries as guideposts to eliminate questions irrelevant for the respondent. For example, only participants who self-reported being assigned female or intersex at birth were asked about whether they retained a cervix. Only participants who said they retained a cervix were asked about cervical cancer screening. Likewise, only participants who self-reported being assigned male or intersex at birth were asked about whether they had a prostate. Only participants who indicated they had a prostate were asked about prostate cancer screening. All participants were asked about breast, colorectal, anal, lung, oral, and skin cancer screening. In addition, participants were asked three open-ended questions, providing an opportunity for respondents to indicate any additional cancer screenings that a provider had recommended, any additional cancer screenings that the participant had received, and recommendations to improve cancer screening experiences for TGNC people.

### Statistical analysis

Descriptive frequencies were used to describe the sample. Logistic regression was performed to generate odds ratios (ORs) for associations between provider recommendation of screenings and actual receipt of screenings for breast, cervical, colorectal, prostate, lung, and anal cancer; in the case of 0 cell values, the Wald-modified OR and confidence limits were calculated.^17^ When comparing provider recommendations and participant-reported screenings, if participants indicated they were not sure if they were recommended for or received a screening, they were dropped from the analysis for each respective logistic regression. One open-text question asked for recommendations from participants to improve the cancer screening experiences of TGNC people.

## Results

Study participants included 58 TGNC people living in the Washington, D.C. area. The age range for the sample was 40–71 years (μ=52.91, standard deviation=8.281). Most participants (*n*=56) reported having a regular health care provider. Of participants assigned female sex at birth, approximately one-third were taking testosterone (*n*=7, 36.8%), with duration ranging from <1 to 10 years of exposure. About one-fifth of participants assigned female sex at birth reported having top surgery (*n*=4, 21.1%), with slightly more reporting a hysterectomy with cervix removal (*n*=6, 31.6%). Of participants assigned male sex at birth, most were taking estrogen (*n*=30, 81.1%), with duration ranging from <1 to 45 years of exposure. One intersex participant reported hormonal therapy with estrogen. Three participants reported having a history of cancer (breast, cervical, Hodgkin's lymphoma, and skin cancer, respectively). Half of participants (*n*=29) indicated a family history of one or more types of cancer: breast (*n*=5), cervical (*n*=3), colorectal (*n*=2), lung (*n*=6), prostate (*n*=5), and other (*n*=14) (Table 1).

**Table 1.** Characteristics of Participants (*n*=58)

Participant characteristics	n (%)^a^
Sex assigned at birth	
Male	37 (63.8)
Female	19 (32.8)
Intersex	2 (3.4)
Race	
American Indian/Alaska Native	4 (6.9)
Asian	2 (3.4)
Black	26 (44.8)
White	27 (46.6)
Ethnicity	
Latino	2 (3.4)
Other	1 (1.7)
Non-Latino	55 (94.8)
Regular health care provider	
Yes	56 (96.6)
No	2 (3.4)
Personal history of cancer	
Yes	3 (5.2)
No	55 (94.8)
Family history of cancer	
Yes	29 (50)
No	28 (48.3)
Not sure	1 (1.7)
Hormonal therapy	
Testosterone (TM)	7 (36.8)^b^
Estrogen (TF)	30 (81.1)^c^
Intersex	1 (50)^d^
Gender-affirming surgery	
Top surgery (TM)	4 (21.1)^b^
Hysterectomy (TM)	6 (31.6)^b^

^a^ Percentage of full sample reported unless otherwise indicated.

^b^ Percentage based on participants assigned female sex at birth.

^c^ Percentage based on participants assigned male sex at birth.

^d^ Percentage based on participants assigned intersex at birth.

TF, respondent on transfeminine spectrum; TM, respondent on transmasculine spectrum.

The primary aim of this study was to examine the relationship between clinical recommendations for screenings and participant receipt of screenings. Provider recommendations were statistically significantly associated with patient receipt of cancer screenings for the following: breast (OR=47.25, 95% confidence interval [CI 9.52–234.47]), colorectal (OR=12.00, 95% CI [3.32–43.42]), prostate (OR=8.00, 95% CI [1.58–40.63]), lung (OR=24.50, 95% CI [2.90–207.29]), and anal (OR=241.57, 95% CI [11.50–5074.71]), all *p*<0.05 (Table 2). Provider recommendation was not significantly associated with patient receipt of cervical cancer screening.

**Table 2.** Effect of Provider Recommendation on Receipt of Screenings

Screening	OR	95% LCL	95% UCL	p^*^
Breast	47.25	9.52	234.47	<0.0001
Cervical (Pap)^a^	8.14	0.26	250.73	0.1185
Colorectal	12.00	3.32	43.42	0.0002
Prostate	8.00	1.58	40.63	0.0121
Lung	24.50	2.90	207.29	0.0033
Anal (UCL)	241.57	11.50	5074.71	<0.0001

^*^ Wald chi-square *p*-value.

^a^ Wald-modified OR and confidence limits due to 0 cell value.

LCL, lower confidence limit; OR, odds ratio; UCL, upper confidence limit.

Of those who should have been recommended for mammography (participants older than 50 years who had not had top surgery if transmasculine or who had been on estrogen for at least 5 years if transfeminine), 87.5% (*n*=7) of transmasculine and 80% (*n*=8) of transfeminine respondents indicated that a provider had recommended mammography in compliance with the University of California Center for Transgender Excellence consensus guidelines for cancer screening.^8^ Of those aged 50 years and older, 68.8% (*n*=22) indicated that a provider had recommended colorectal cancer screening.

For cervical cancer, only 64% of respondents (*n*=9) who retained a cervix (*n*=14) were told by their providers to get screened for cervical cancer. Participants were asked their preference for self-swab versus clinician-administered swab for HPV cotesting when screened for cervical cancer. Responses were mixed, with four respondents indicating a preference for self-swab and ten indicating a preference for clinician-administered swab. Only participants with a cervix answered this question.

Participants were also asked about receipt of oral and skin cancer screenings, but were not asked about whether a provider recommended these screening, since the USPSTF indicates inconclusive evidence for the utility of these screenings.^13,14^ Nineteen respondents indicated having received an oral cancer screening in the past, ranging from <1 to 5 years ago. Twenty participants reported having been screened for skin cancer, ranging from <1 to 10 years ago.

An open-ended question asked respondents for suggestions on how to improve cancer screening experiences for TGNC people. Feedback indicated the need for improved interpersonal skills on the part of health care professionals: “Doctors should understand and use non-binary pronouns and understand the difference between sex, gender, presentation, and orientation.” A theme in respondent feedback was the need for relevant information: “Make us aware of what we should be screened for” and “suggest [screening] with other examinations.” Finally, cues of safety were mentioned as important: “I am more likely to set an appointment and to trust a provider that states on its website that it affirms gender non-conforming people.”

## Discussion

The present study is the first known study of cancer screening experiences of TGNC people. The study is important, because provider recommendations were statistically significantly associated with patient screening behaviors in this sample for breast, colorectal, prostate, lung, and anal cancer. No statistically significant results were found for cervical cancer screening: more respondents initiated cervical cancer screening than reported a provider recommending the screening. This result is counter to past studies to date.^7^ This pattern was reversed for anal cancer screening: fewer people received anal cancer screening compared with provider recommendations for screening. However, those who were recommended for anal cancer screening were more than twice as likely to be screened versus those who had not received a recommendation.

There are several important things to consider when interpreting these results. First, the sample is not a representative sample; therefore, a comparison of cancer screening behaviors among transgender participants in this sample with population-based cisgender samples is not likely to yield meaningful information.

Second, it is critical to note that while this article reports the association between provider recommendations and self-report of cancer screenings in an urban sample, not all screenings are recommended in average-risk people. Screening for cancer when it is not necessary can lead to false positives, which may lead to unnecessary psychological distress,^18^ unnecessary tests and procedures, and unnecessary individual and societal health care costs. For example, while provider recommendations were associated with self-reported screening behaviors for prostate cancer in this study, guidelines indicate that prostate cancer screening is not universally recommended for those with a prostate at average risk. The USPSTF provides a C grade for prostate cancer screening, recommending that clinicians not screen individuals unless they “express a preference for screening.”^19^ Likewise, lung cancer screening is only indicated for people older than 55 years with a 30 pack-year history,^5,11^ and there is no consensus for anal cancer screening within the United States. Furthermore, the USPSTF considers evidence for skin screening inconclusive.^13,14^

Taken together, there is great onus on individual providers to assess individual risks of patients and to recommend cancer screenings accordingly. Previous studies have found that physician reminders are significantly associated with physician screening recommendations; thus, electronic reminders may be one way to increase provider attention to indicated cancer screenings.^20^ Providers likely vary widely in their recommendations for patients with similar risks due to a dearth of evidence-based guidance. To remedy this, large cancer screening studies will need to analyze the heterogeneity of screening effects on people that vary based on sex, gender identity, endogenous/exogenous hormone balance, body composition and anatomy, as well as other sociodemographic factors. The evidence in this study suggests that provider recommendation matters for TGNC individuals. It is critical that research catch up with clinical demands to provide better evidence to inform screening recommendations to ensure lifesaving early detection while avoiding unnecessary physical, psychological, and financial costs of overscreening.

### Strengths and limitations

A significant strength of this study is the diversity of race in the sample. Limitations of the study include its relatively small sample size and its cross-sectional design, preventing longitudinal follow-up of the sample. Convenience sampling may also lead to nonrandom sampling bias. Sampling bias is likely, given the method of recruitment at transgender-focused community events. Individuals less likely to come out to a social event or less socially connected with other transgender people were less likely to be offered survey participation in this study. Self-report data are also subject to social desirability and recall biases. Future studies should recruit larger sample sizes in diverse geographic areas and collect additional demographic information to allow for subgroup analyses. In addition, the study did not collect information about history of tobacco use, HIV status, or anal/oral-receptive sexual behaviors. These additional data would have helped further contextualize study findings. Finally, the survey asked respondents to confirm identification along the transgender spectrum, but did not ask for greater details—thus, the use of the terms “transfeminine” and “transmasculine” in this article refers to the spectrum of trans experiences. Future refinements of the survey instrument should include more granular data.

## Conclusion

This study presents evidence that provider recommendations are associated with TGNC breast, colorectal, prostate, lung, and anal cancer screening adherence. This study also suggests that there are opportunities to strengthen provider recommendations for routine cancer screenings, especially colorectal and cervical cancer screenings. More research is needed to inform optimal clinical recommendations for TGNC cancer continuum of care services. Inclusion of gender identity, anatomy, and hormone exposure in future cancer screening research studies will provide data to inform provider recommendations based on population-level and individual-level risks of TGNC people.

## Supplementary Material

Supplemental data

## Abbreviations Used

CI: confidence interval
IRB: Institutional Review Board
LCL: lower confidence limit
OR: odds ratio
TF: respondent on transfeminine spectrum
TGNC: transgender and gender nonconforming
TM: respondent on transmasculine spectrum
UCL: upper confidence limit
USPSTF: U.S. Preventive Services Task Force

## References

[1] Dubin SN, Nolan IT, Streed CG Jr., et al. Transgender health care: improving medical students' and residents' training and awareness. Adv Med Educ Pract 2018;9:377–391 29849472 10.2147/AMEP.S147183 PMC5967378

[2] Morrison SD, Wilson SC, Smith JR Are we adequately preparing our trainees to care for transgender patients? J Grad Med Educ 2017;9:258 28439369 10.4300/JGME-D-16-00712.1 PMC5398125

[3] Khalili J, Leung LB, Diamant AL Finding the perfect doctor: identifying lesbian, gay, bisexual, and transgender-competent physicians. Am J Public Health 2015;105:1114–1119 25880937 10.2105/AJPH.2014.302448 PMC4431087

[4] Grant JM, Mottet LA, Tanis J. National Transgender Discrimination Survey report on Health and Health Care. 2010. Washington, DC, National Center for Transgender Equality and the National Gay and Lesbian Task Force

[5] Pratt-Chapman ML, Potter J Cancer care considerations for sexual and gender minority patients. Oncol Issues 2019;36:26–36

[6] de Block CJM, Wiepjes C, Nota NM, et al. Breast cancer risk in transgender people receiving hormone treatment: nationwide cohort study in the Netherlands. BMJ 2019;365:1652 10.1136/bmj.l1652 PMC6515308 31088823

[7] Peitzmeier SM, Khullar K, Reisner SL, Potter J Pap test use is lower among female-to-male patients than non-transgender women. Am J Prev Med 2014;47:808–812 25455121 10.1016/j.amepre.2014.07.031

[8] American Cancer Society. Cancer screening guidelines. Available at https://www.cancer.org/healthy/find-cancer-early/cancer-screening-guidelines.html Accessed 915, 2019

[9] Duetsch MB, ed: UCSF Transgender Care. Guidelines for the primary and gender-affirming care of transgender and gender nonbinary people. Available at transcare.ucsf.edu/guidelines Accessed 915, 2019

[10] U.S. Preventive Services Task Force. Final recommendation statement: colorectal cancer: screening. Available at uspreventiveservicestaskforce.org/Page/Document/RecommendationStatementFinal/colorectal-cancer-screening2 Accessed 915, 2019

[11] U.S. Preventive Services Task Force. Final recommendation statement: lung cancer: screening. Available at uspreventiveservicestaskforce.org/Page/Document/RecommendationStatementFinal/lung-cancer-screening Accessed 915, 2019

[12] Glynne-Jones R, Nilsson PJ, Aschele C, et al. Anal cancer: ESMO-ESSO-ESTRO Clinical Practice Guidelines for diagnosis, treatment and follow-up. Ann Oncol 2014;25(Suppl. 3):iii10–iii20 25001200 10.1093/annonc/mdu159

[13] U.S. Preventive Services Task Force. Skin cancer screening. Available at https://www.uspreventiveservicestaskforce.org/Page/Document/UpdateSummaryFinal/skin-cancer-screening2 Accessed 131, 2020

[14] U.S. Preventive Services Task Force. Oral cancer screening. Available at https://www.uspreventiveservicestaskforce.org/Page/Document/RecommendationStatementFinal/oral-cancer-screening1 Accessed 131, 2020

[15] National Institutes of Health Sexual and Gender Minority Research Office. Available at https://dpcpsi.nih.gov/sgmro Accessed 915, 2019

[16] Griggs J, Maingi S, Blinder V, et al. American Society of Clinical Oncology position statement: strategies for reducing cancer health disparities among sexual and gender minority populations. J Clin Oncol 2017;35:2203–2208 28368670 10.1200/JCO.2016.72.0441

[17] Haldane JBS The estimation and significance of the logarithm of a ratio of frequencies. Ann Hum Genet 1955;20:309–314 10.1111/j.1469-1809.1955.tb01285.x 13314400

[18] Kroenke K Are the harms of false-positive screening test results minimal or meaningful? JAMA Netw 2014;174:961–963 10.1001/jamainternmed.2014.160 24756378

[19] U.S. Preventive Services Task Force. Final recommendation statement: prostate cancer: screening. Available at https://www.uspreventiveservicestaskforce.org/Page/Document/RecommendationStatementFinal/prostate-cancer-screening1 Accessed 26, 2020

