# Supplementary material for: Provider Recommendations Are Associated with Cancer Screening of Transgender and Gender-Nonconforming People: A Cross-Sectional Urban Survey
Source: Transgend Health. 2020 Jun 8;5(2):80–5. doi: 10.1089/trgh.2019.0083 (PMC7347019; doi:10.1089/trgh.2019.0083)
Supplement: Supplemental data [file Supp_Data.doc]

**Supplementary Data**

**Supplementary Appendix S1. Transgender and Gender-Nonconforming Cancer Screening Survey**

| **No.** | **Item** | **Answer options** |
| --- | --- | --- |
| Screening 1 | Are you AT LEAST 40 years of age? | | 1 | Yes | | --- | --- | | 0 | No |   If no, terminate survey |
| Screening 2 | This survey is intended for people who are transgender, genderqueer, gender nonconforming, and/or gender nonbinary. There are many more terms that are not listed here that we would consider included under the umbrella of “gender nonconforming.”  Do you consider yourself anywhere along the transgender or gender-nonconforming spectrum? | | 1 | Yes | | --- | --- | | 0 | No |   If no, terminate survey |
| Screening 3 | Do you live in Washington, D.C., Maryland, or Virginia? | | 1 | Yes | | --- | --- | | 0 | No |   If no, terminate survey |
| Screening 4 | Do you agree to provide your true experiences as you complete this survey? | | 1 | Yes | | --- | --- | | 0 | No |   If no, terminate survey |
| 1a | I am completing this survey at the following event: | | 1 | April 27 Whitman-Walker Health’s Gender Affirming Open House | | --- | --- | | 2 | May 18 Trans PRIDE | | 3 | June 9 Capitol PRIDE | | 4 | Phone call with GW staff | | 5 | Some other event (please specify) | |
| 1b | If other, where are you taking this survey? | Open text |
| 2 | Please indicate the sex you were assigned at birth.  “Assigned at birth” means the sex the doctor said should be on your original birth certificate | | 1 | Female | | --- | --- | | 2 | Male | | 3 | Intersex or X | |
| 3 | How old are you? | Integer |
| 4 | Which categories describe you? (Select all that apply) | | 1 | American Indian or Alaska Native | | --- | --- | | 2 | Asian | | 3 | Black or African American | | 4 | Hispanic or Latinx | | 5 | Native Hawaiian or other Pacific Islander | | 6 | White | | 7 | Other | |
| 5 | If other, please specify: | Open text |
| 6 | Do you have a health care provider or clinic that you go to for regular health care? | | 1 | Yes | | --- | --- | | 0 | No | |
| 7 | Have you ever been diagnosed with cancer? | | 1 | Yes | | --- | --- | | 0 | No | | 2 | I’m not sure | |
| 8a | Which types of cancer have you been diagnosed with? (Select all that apply) | | 1 | Anal | | --- | --- | | 2 | Breast | | 3 | Cervical | | 4 | Colorectal | | 5 | Lung | | 6 | Prostate | | 7 | Other | |
| 8b | Please specify the cancer type: | Open text |
| 9 | Have your parents, siblings, or children ever been diagnosed with cancer? | | 1 | Yes | | --- | --- | | 0 | No | | 2 | I’m not sure | |
| 10a | Which types of cancer have your parents, siblings, or children had? (Select all that apply) | | 1 | Anal | | --- | --- | | 2 | Breast | | 3 | Cervical | | 4 | Colorectal | | 5 | Lung | | 6 | Prostate | | 7 | Other | |
| 10b | Please specify the cancer type: | Open text |
| 11 | Has a health care professional ever told you that you should be screened for breast cancer? | | 1 | Yes | | --- | --- | | 0 | No | | 2 | I’m not sure | |
| 12 | Breast cancer screening usually starts with a mammogram. A mammogram is when your breasts are examined using a low-dose X-ray machine. Sometimes a breast MRI is done after a mammogram or instead of a mammogram. Breast screening does NOT include a clinical breast examination (when a health care professional examines your breasts with their hands).  Have you EVER been screened for breast cancer? | | 1 | Yes | | --- | --- | | 0 | No | | 2 | I’m not sure | |
| 13 | No. of years since your most recent breast screening:  *Please round to the closest number of years. If less than a year, indicate 0* | Integer |
| N/A | Section Header: Intersex people have a variety of combinations of sex organs and may or may not be on hormonal therapy | N/A |
| 14 | Do you currently take or have you ever taken testosterone? | | 1 | Yes | | --- | --- | | 0 | No | |
| 15 | For how many years in total have you taken testosterone?  *Please round to the closest number of years. If less than a year, indicate 0* | Integer |
| 16 | Have you had top surgery? | | 1 | Yes | | --- | --- | | 0 | No | |
| 17 | No. of years since your top surgery:  *Please round to the closest number of years. If less than a year, indicate 0* | Integer |
| 18 | People assigned female at birth have a cervix unless they have had a hysterectomy (removal of the uterus) that included removing the cervix.  Do you have a cervix? | | 1 | Yes | | --- | --- | | 0 | No | | 2 | I’m not sure | |
| 19 | No. of years since you had your cervix removed:  *Please round to the closest number of years. If less than a year, indicate 0* | Integer |
| 20 | Has a health care professional ever told you that you should be screened for cervical cancer? | | 1 | Yes | | --- | --- | | 0 | No | | 2 | I’m not sure | |
| 21 | Cervical cancer screening usually involves a speculum being inserted into your front hole or vagina. This is called a Pap test or Pap smear.  Have you EVER had a Pap test or Pap smear? | | 1 | Yes | | --- | --- | | 0 | No | | 2 | I’m not sure | |
| 22 | No. of years since your most recent Pap test or Pap smear:  *Please round to the closest number of years. If less than a year, indicate 0* | Integer |
| 23 | Another form of cervical cancer screening is an HPV test. HPV stands for human papillomavirus. This usually involves taking a sample from the cervix at the same time as a Pap test.  Have you EVER had an HPV test? | | 1 | Yes | | --- | --- | | 0 | No | | 2 | I’m not sure | |
| 24 | No. of years since your most recent HPV test:  *Please round to the closest number of years. If less than a year, indicate 0* | Integer |
| 25 | If you had the option, would you prefer a doctor to check your cervix or would you prefer the option to use a cotton swab to take your own sample and give it to your health care provider? | | 1 | I would rather have a doctor check my cervix | | --- | --- | | 2 | I would rather self-sample | | 3 | I do not have a preference | |
| 26 | Are you currently taking or have you ever taken estradiol or any form of estrogen? | | 1 | Yes | | --- | --- | | 0 | No | |
| 27 | Please indicate for how many years in total you have taken any form of estrogen.  *Please round to the closest number of years. If less than a year, indicate 0* | Integer |
| 28 | Do you have a prostate? | | 1 | Yes | | --- | --- | | 0 | No | | 2 | I’m not sure | |
| 29 | Has a health care professional ever told you that you should be screened for prostate cancer?  Prostate screening involves a blood test called the PSA test. It also involves having a provider examine your prostate by putting a gloved finger in your rectum or anus. This is called a digital rectal examination or DRE. If you have a vagina, your health care provider can also examine your prostate using a vaginal ultrasound | | 1 | Yes | | --- | --- | | 0 | No | | 2 | I’m not sure | |
| 30 | Have you EVER been screened for prostate cancer? | | 1 | Yes | | --- | --- | | 0 | No | |
| 31 | No. of years since your most recent prostate cancer screening:  *Please round to the closest number of years. If less than a year, indicate 0* | Integer |
| 32 | Section Header: Have you received an HPV vaccine? | | 1 | Yes | | --- | --- | | 0 | No | | 2 | I’m not sure | |
| 33 | A full treatment to prevent HPV requires 2–3 shots. How many shots did you receive? | | 1 | 1 | | --- | --- | | 2 | 2 | | 3 | 3 | | 4 | I’m not sure | |
| 34 | Has a health care professional ever told you that you should be screened for colorectal cancer? | | 1 | Yes | | --- | --- | | 0 | No | | 2 | I’m not sure | |
| 35 | There are many ways to be screened for colorectal cancer. Colonoscopy and flexible sigmoidoscopy involve having a flexible tube inserted into your anus. An FIT or FOBT test involves getting a fecal (poop) sample and sending that to be tested by your health care provider.  Have you EVER had colorectal cancer screening? | | 1 | Yes | | --- | --- | | 0 | No | | 2 | I’m not sure | |
| 36 | What type of colorectal screening did you have? | | 1 | Colonoscopy | | --- | --- | | 2 | Flexible sigmoidoscopy | | 3 | FIT or FOBT test | |
| 37 | No. of years since your most recent colorectal screening:  *Please round to the closest number of years. If less than a year, indicate 0* | Integer |
| 38 | Has your health care provider ever recommended that you be screened for lung cancer? | | 1 | Yes | | --- | --- | | 0 | No | | 2 | I’m not sure | |
| 39 | Lung cancer screening involves something called a low-dose CT scan, because it uses low-dose X-rays and computer imaging.  Have you EVER had a lung cancer screening? | | 1 | Yes | | --- | --- | | 0 | No | | 2 | I’m not sure | |
| 40 | No. of years since your most recent lung cancer screening:  *Please round to the closest number of years. If less than a year, indicate 0* | Integer |
| 41 | Has your health care provider ever recommended that you be screened for anal cancer? | | 1 | Yes | | --- | --- | | 0 | No | | 2 | I’m not sure | |
| 42 | Anal cancer screening involves an anal Pap test.  Have you EVER had anal cancer screening? | | 1 | Yes | | --- | --- | | 0 | No | | 2 | I’m not sure | |
| 43 | No. of years since your most recent anal cancer screening:  *Please round to the closest number of years. If less than a year, indicate 0* | Integer |
| 44 | Oral cancer screening happens when a doctor or dentist looks in your mouth and examines your tongue.  Have you EVER been screened for oral cancer? | | 1 | Yes | | --- | --- | | 0 | No | | 2 | I’m not sure | |
| 45 | No. of years since you were last examined for oral cancer:  *Please round to the closest number of years. If less than a year, indicate 0* | Integer |
| 46 | Skin cancer screening is when a doctor examines your full body for unusual moles or skin changes.  Has a doctor EVER examined your body for skin cancer? | | 1 | Yes | | --- | --- | | 0 | No | | 2 | I’m not sure | |
| 47 | No. of years since you were last examined for skin cancer:  *Please round to the closest number of years. If less than a year, indicate 0* | Integer |
| 48 | Please indicate any other cancer screenings that have been recommended by your health care provider | Open text |
| 49 | Please indicate any other cancer screenings that you have had | Open text |
| 50 | Please offer any suggestions on how to improve the cancer screening process for transgender and gender-nonconforming people | Open text |
